# Supplementary material for: Mediterranean Diet to Prevent the Development of Colon Diseases: A Meta-Analysis of Gut Microbiota Studies
Source: Nutrients. 2021 Jun 29;13(7):2234. doi: 10.3390/nu13072234 (PMC8308215; doi:10.3390/nu13072234)
Supplement: Supplementary file 1 [file nutrients-13-02234-s001.zip › Supplemental/Supplemental figures.pdf]

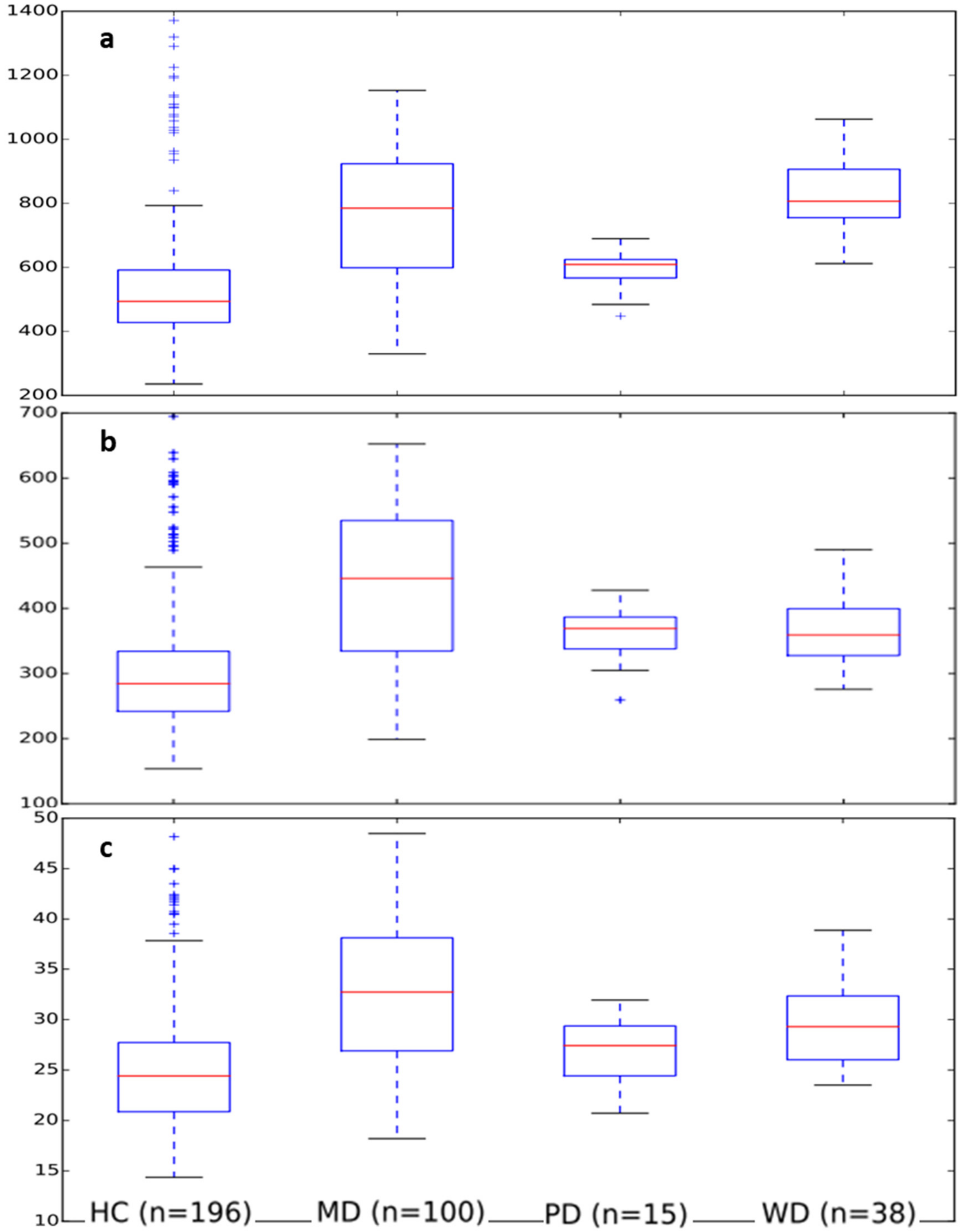

Figure S1. Alpha diversity comparison between diets. Comparison of healthy subjects following a non-defined diet (HC), Mediterranean diet (MD), Modern paleolithic diet (PD) or Western-type diet (WD). Alpha diversity metrics used were **a)** Chao1, **b)** total observed OTUs and **c)** phylogenetic distance.

Table S8. Alpha diversity iet groups pairings comparisons. Non-parametric T-test results.

Metric	Group1	Group2	Group1 mean	Group1 stdev	Group2 mean	Group2 stdev	t stat	p-value*	p-value**
Chao1	PD	HC	590.74	66.61	560.73	222.87	0.517	1	0.609
	WD	HC	826.14	112.10	560.73	222.87	7.137	0.006	0.006
	MD	PD	767.15	198.06	590.74	66.61	3.391	0.006	0.003
	MD	HC	767.15	198.06	560.73	222.87	7.793	0.006	0.003
	WD	PD	826.14	112.10	590.74	66.61	7.474	0.006	0.002
	MD	WD	767.15	198.06	826.14	112.10	-1.721	0.552	0.104
Observed OTUs	PD	HC	361.55	43.86	309.95	108.11	1.828	0.414	0.086
	WD	HC	360.41	48.47	309.95	108.11	2.811	0.024	0.01
	MD	PD	439.23	123.85	361.55	43.86	2.386	0.144	0.021
	MD	HC	439.23	123.85	309.95	108.11	9.223	0.006	0.006
	WD	PD	360.41	48.47	361.55	43.86	-0.078	1	0.945
	MD	WD	439.23	123.85	360.41	48.47	3.786	0.006	0.003
Phylogenetic distance	PD	HC	27.26	3.28	25.45	6.75	1.022	1	0.333
	WD	HC	29.59	3.80	25.45	6.75	3.646	0.018	0.006
	MD	PD	32.66	7.64	27.26	3.28	2.677	0.024	0.012
	MD	HC	32.66	7.64	25.45	6.75	8.274	0.006	0.006
	WD	PD	29.59	3.80	27.26	3.28	2.046	0.312	0.050
	MD	WD	32.66	7.64	29.59	3.80	2.356	0.144	0.026

*Bonferroni corrected. **False discovery rate corrected.

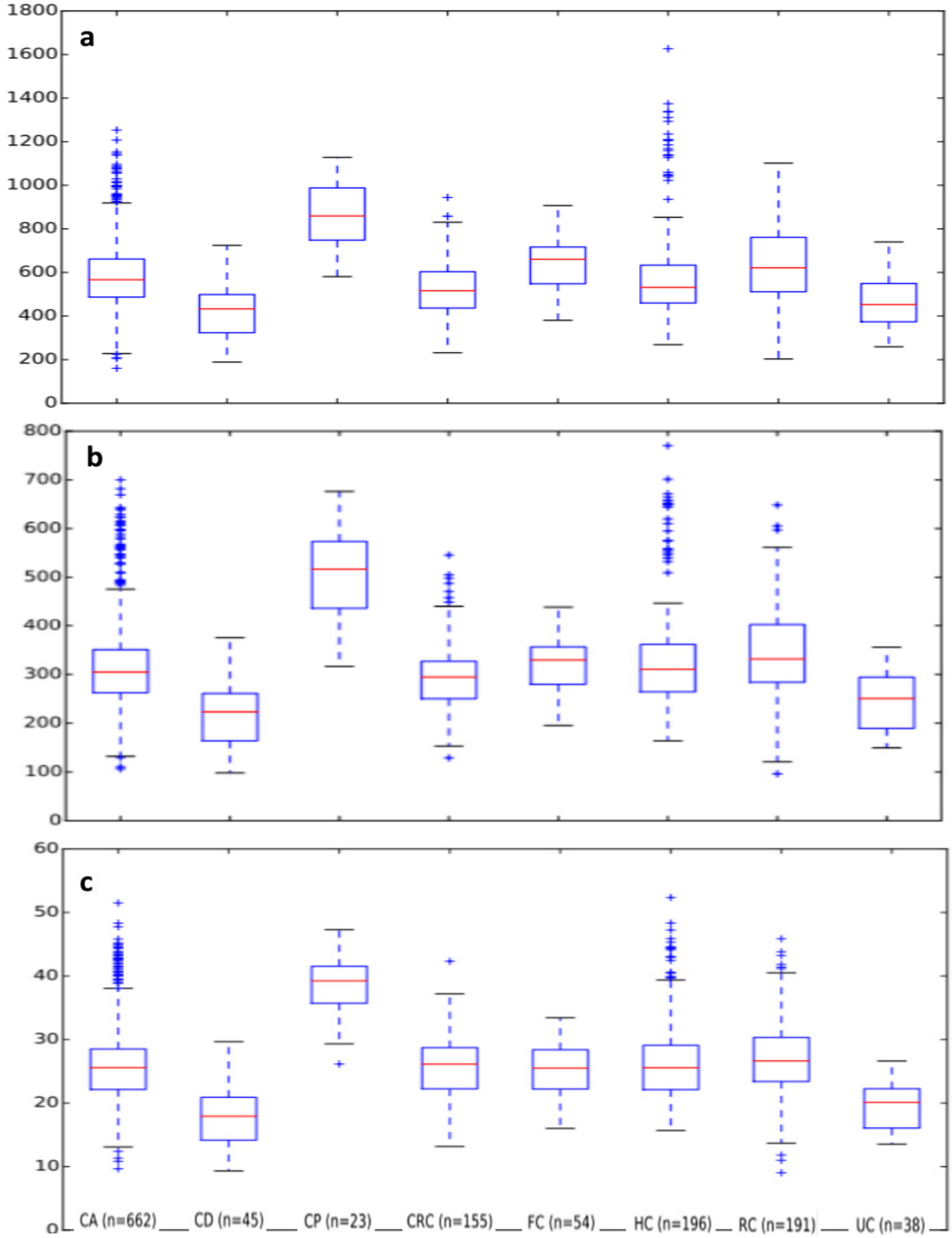

Figure S2. Alpha diversity comparison between diseases. Comparison of healthy subjects (HC), at-risk controls (RC), IBD patients familiars (FC), colon polyposis (CP), colon adenomatosis (CA), colorectal cancer (CRC), ulcerative colitis (UC) and Crohn's disease (CD) patients. Alpha diversity metrics used were **a)** Chao1, **b)** total observed OTUs and **c)** phylogenetic distance.

Table S9. Alpha diversity disease groups pairings comparisons. Non-parametric T-test results.

Metric	Group1	Group2	Group1 mean	Group1 stdev	Group2 mean	Group2 stdev	t stat	p-value*	p-value**
Chao1	CD	CP	420.07	125.92	860.43	151.77	-12.517	0.028	0.028
	CD	HC	420.07	125.92	603.73	241.45	-4.930	0.028	0.002
	CD	UC	420.07	125.92	462.23	115.27	-1.560	1	0.138
	CA	FC	586.16	163.27	636.52	114.10	-2.219	0.84	0.037
	CA	CD	586.16	163.27	420.07	125.92	6.681	0.028	0.002
	CA	UC	586.16	163.27	462.23	115.27	4.607	0.028	0.003
	CRC	HC	523.50	128.77	603.73	241.45	-3.727	0.028	0.002
	CRC	RS	523.50	128.77	632.42	170.82	-6.549	0.028	0.002
	FC	RS	636.52	114.10	632.42	170.82	0.165	1	0.857
	CRC	CP	523.50	128.77	860.43	151.77	-11.362	0.028	0.002
	CD	CRC	420.07	125.92	523.50	128.77	-4.743	0.028	0.001
	FC	CP	636.52	114.10	860.43	151.77	-7.014	0.028	0.003
	CA	HC	586.16	163.27	603.73	241.45	-1.172	1	0.251
	FC	HC	636.52	114.10	603.73	241.45	0.965	1	0.371
	CA	CP	586.16	163.27	860.43	151.77	-7.927	0.028	0.003
	CA	RS	586.16	163.27	632.42	170.82	-3.409	0.028	0.003
	CD	FC	420.07	125.92	636.52	114.10	-8.874	0.028	0.009
	CRC	FC	523.50	128.77	636.52	114.10	-5.688	0.028	0.002
	HC	UC	603.73	241.45	462.23	115.27	3.520	0.028	0.003
	CD	RS	420.07	125.92	632.42	170.82	-7.818	0.028	0.004
	CA	CRC	586.16	163.27	523.50	128.77	4.459	0.028	0.004
	CRC	UC	523.50	128.77	462.23	115.27	2.667	0.308	0.011
	HC	CP	603.73	241.45	860.43	151.77	-4.962	0.028	0.006
	CP	UC	860.43	151.77	462.23	115.27	11.382	0.028	0.007
	RS	CP	632.42	170.82	860.43	151.77	-6.089	0.028	0.005
	FC	UC	636.52	114.10	462.23	115.27	7.105	0.028	0.014
	RS	HC	632.42	170.82	603.73	241.45	1.343	1	0.199
	RS	UC	632.42	170.82	462.23	115.27	5.855	0.028	0.002
Observed OTUs	CD	CP	211.57	62.84	502.25	92.90	-15.021	0.028	0.028
	CD	HC	211.57	62.84	336.92	118.19	-6.865	0.028	0.001
	CD	UC	211.57	62.84	242.73	59.39	-2.280	0.812	0.028
	CA	FC	318.53	93.51	320.37	54.27	-0.142	1	0.884
	CA	CD	318.53	93.51	211.57	62.84	7.547	0.028	0.002
	CA	UC	318.53	93.51	242.73	59.39	4.933	0.028	0.003
	CRC	HC	297.58	72.11	336.92	118.19	-3.633	0.028	0.002
	CRC	RS	297.58	72.11	344.83	94.97	-5.098	0.028	0.002
	FC	RS	320.37	54.27	344.83	94.97	-1.803	1	0.072
	CRC	CP	297.58	72.11	502.25	92.90	-12.124	0.028	0.002
	CD	CRC	211.57	62.84	297.58	72.11	-7.206	0.028	0.002
	FC	CP	320.37	54.27	502.25	92.90	-10.580	0.028	0.001
	CA	HC	318.53	93.51	336.92	118.19	-2.266	0.728	0.028
	FC	HC	320.37	54.27	336.92	118.19	-0.997	1	0.352
	CA	CP	318.53	93.51	502.25	92.90	-9.252	0.028	0.003
	CA	RS	318.53	93.51	344.83	94.97	-3.408	0.028	0.003
	CD	FC	211.57	62.84	320.37	54.27	-9.148	0.028	0.009

*Bonferroni corrected. **False discovery rate corrected.

Table S9. Alpha diversity disease groups pairings comparisons. Non-parametric T-test results. (Continuation)

	Group1	Group2	Group1 mean	Group1 stdev	Group2 mean	Group2 stdev	t stat	p-value*	p-value**
Observed OTUs	CRC	FC	297.58	72.11	320.37	54.27	-2.112	1	0.043
	HC	UC	336.92	118.19	242.73	59.39	4.776	0.028	0.004
	CD	RS	211.57	62.84	344.83	94.97	-8.923	0.028	0.004
	CA	CRC	318.53	93.51	297.58	72.11	2.610	0.392	0.013
	CRC	UC	297.58	72.11	242.73	59.39	4.319	0.028	0.002
	HC	CP	336.92	118.19	502.25	92.90	-6.448	0.028	0.006
	CP	UC	502.25	92.90	242.73	59.39	13.085	0.028	0.007
	RS	CP	344.83	94.97	502.25	92.90	-7.492	0.028	0.005
	FC	UC	320.37	54.27	242.73	59.39	6.426	0.028	0.014
	RS	HC	344.83	94.97	336.92	118.19	0.722	1	0.465
	RS	UC	344.83	94.97	242.73	59.39	6.355	0.028	0.002
Phylogenetic distance	CD	CP	17.63	4.68	38.02	5.26	-16.058	0.028	0.028
	CD	HC	17.63	4.68	26.83	7.19	-8.162	0.028	0.002
	CD	UC	17.63	4.68	19.73	3.86	-2.182	0.924	0.045
	CA	FC	26.16	6.30	25.42	4.34	0.847	1	0.473
	CA	CD	26.16	6.30	17.63	4.68	8.898	0.028	0.002
	CA	UC	26.16	6.30	19.73	3.86	6.208	0.028	0.003
	CRC	HC	25.88	4.93	26.83	7.19	-1.400	1	0.201
	CRC	RS	25.88	4.93	27.05	6.25	-1.905	1	0.080
	FC	RS	25.42	4.34	27.05	6.25	-1.800	1	0.081
	CRC	CP	25.88	4.93	38.02	5.26	-10.859	0.028	0.002
	CD	CRC	17.63	4.68	25.88	4.93	-9.941	0.028	0.002
	FC	CP	25.42	4.34	38.02	5.26	-10.789	0.028	0.003
	CA	HC	26.16	6.30	26.83	7.19	-1.262	1	0.253
	FC	HC	25.42	4.34	26.83	7.19	-1.370	1	0.234
	CA	CP	26.16	6.30	38.02	5.26	-8.902	0.028	0.003
	CA	RS	26.16	6.30	27.05	6.25	-1.733	1	0.112
	CD	FC	17.63	4.68	25.42	4.34	-8.499	0.028	0.009
	CRC	FC	25.88	4.93	25.42	4.34	0.607	1	0.569
	HC	UC	26.83	7.19	19.73	3.86	5.897	0.028	0.004
	CD	RS	17.63	4.68	27.05	6.25	-9.474	0.028	0.004
	CA	CRC	26.16	6.30	25.88	4.93	0.517	1	0.629
	CRC	UC	25.88	4.93	19.73	3.86	7.126	0.028	0.002
	HC	CP	26.83	7.19	38.02	5.26	-7.210	0.028	0.006
	CP	UC	38.02	5.26	19.73	3.86	15.341	0.028	0.007
	RS	CP	27.05	6.25	38.02	5.26	-8.043	0.028	0.005
	FC	UC	25.42	4.34	19.73	3.86	6.407	0.028	0.014
	RS	HC	27.05	6.25	26.83	7.19	0.330	1	0.765
	RS	UC	27.05	6.25	19.73	3.86	6.937	0.028	0.002

*Bonferroni corrected. **False discovery rate corrected.

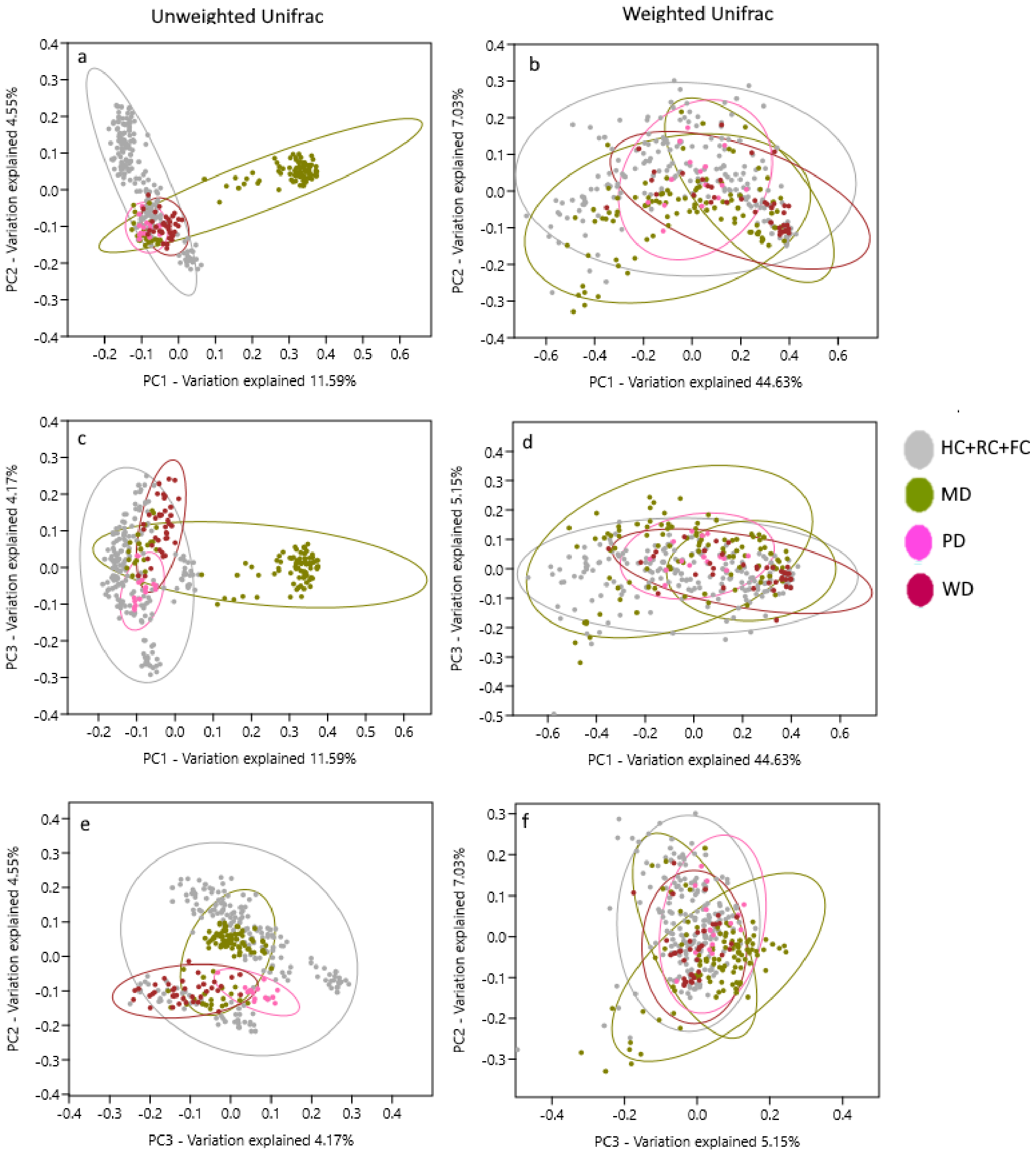

Figure S3. Beta diversity principal coordinates (PCoA) analysis of diets. Comparison of healthy subjects following a non-defined diet (HC+RC+FC), a Mediterranean diet (MD), a modern paleolithic diet (PD) or a western-type diet (WD). The Unifrac distance metric either unweighted (**a**, **c**, **e**) and weighted (**b**, **d**, **f**) was used. Ellipses indicate 95% confidence intervals.

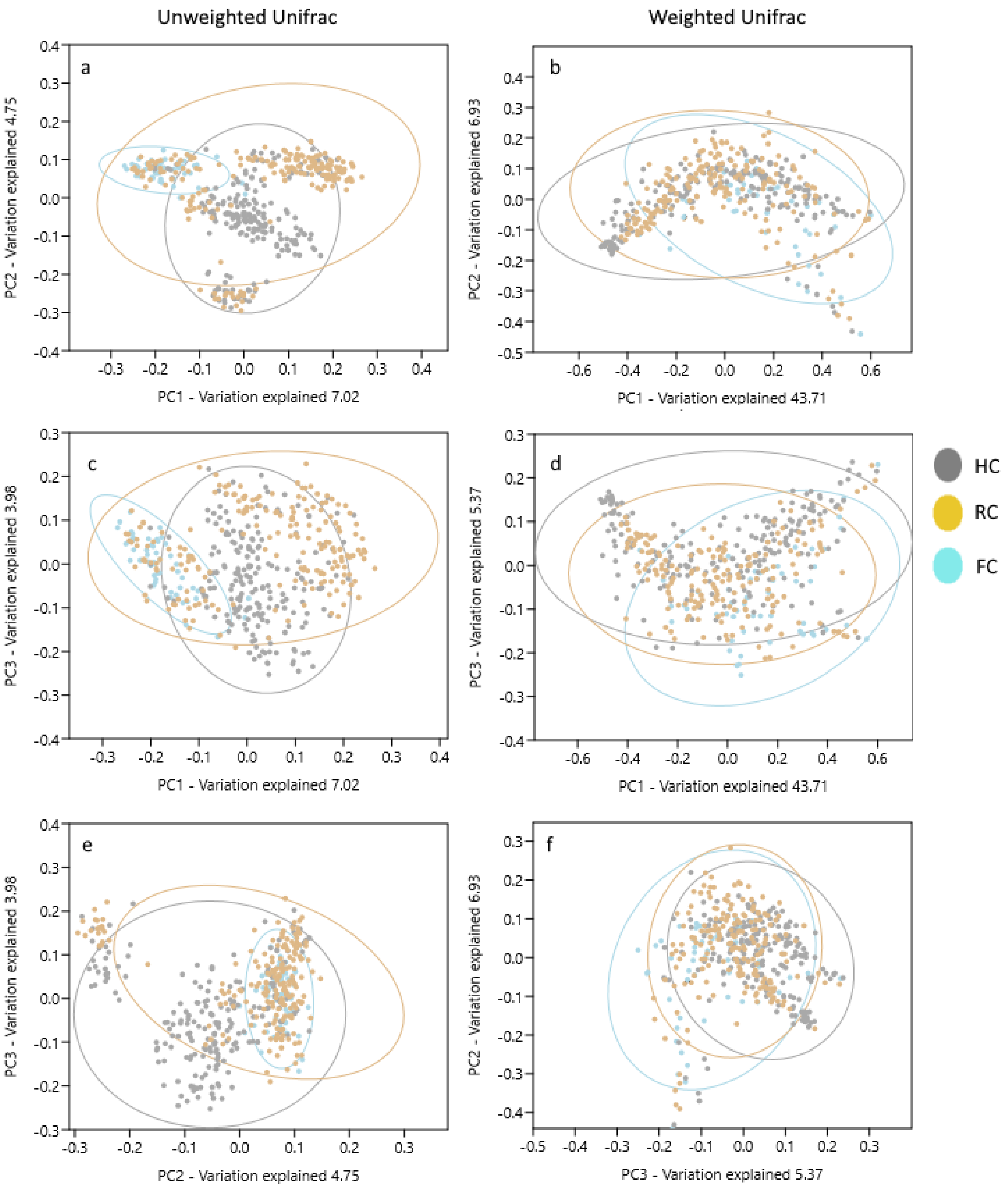

Figure S4. Beta diversity principal coordinates (PCoA) analysis of control groups. Comparison of healthy subjects related (FC) or non-related to IBD patients (HC), and non-related individuals at risk of developing metabolic diseases (RC). The Unifrac distance metric either unweighted (**a**, **c**, **e**) and weighted (**b**, **d**, **f**) was used. Ellipses indicate 95% confidence intervals.

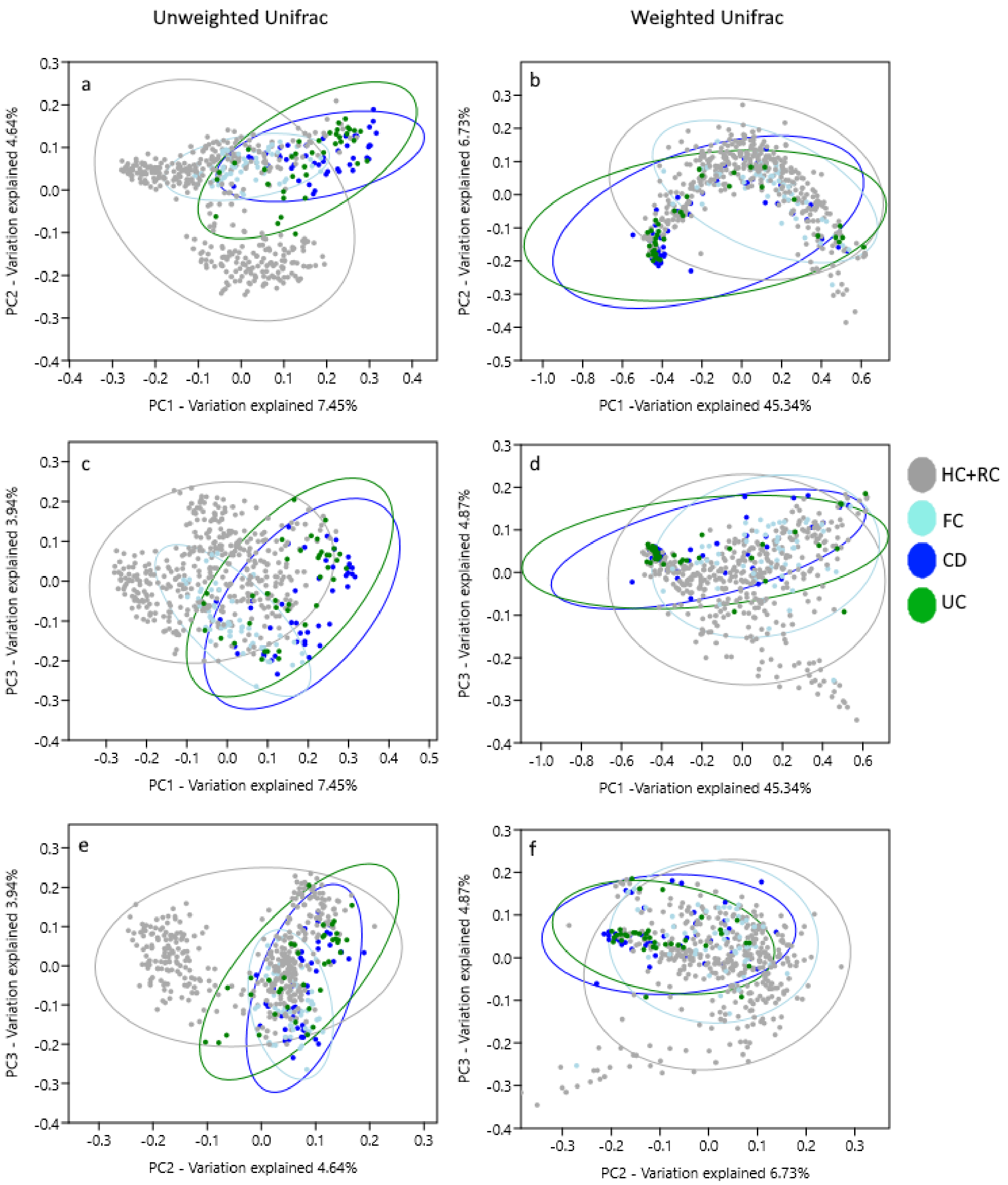

Figure S5. Beta diversity principal coordinates (PCoA) analysis of IBD groups. Comparison of healthy subjects related (FC) or non-related to IBD patients (HC+RC), and Crohn's disease (CD) or ulcerative colitis (UC) patients. The Unifrac distance metric either unweighted (**a**, **c**, **e**) and weighted (**b**, **d**, **f**) was used. Ellipses indicate 95% confidence intervals.

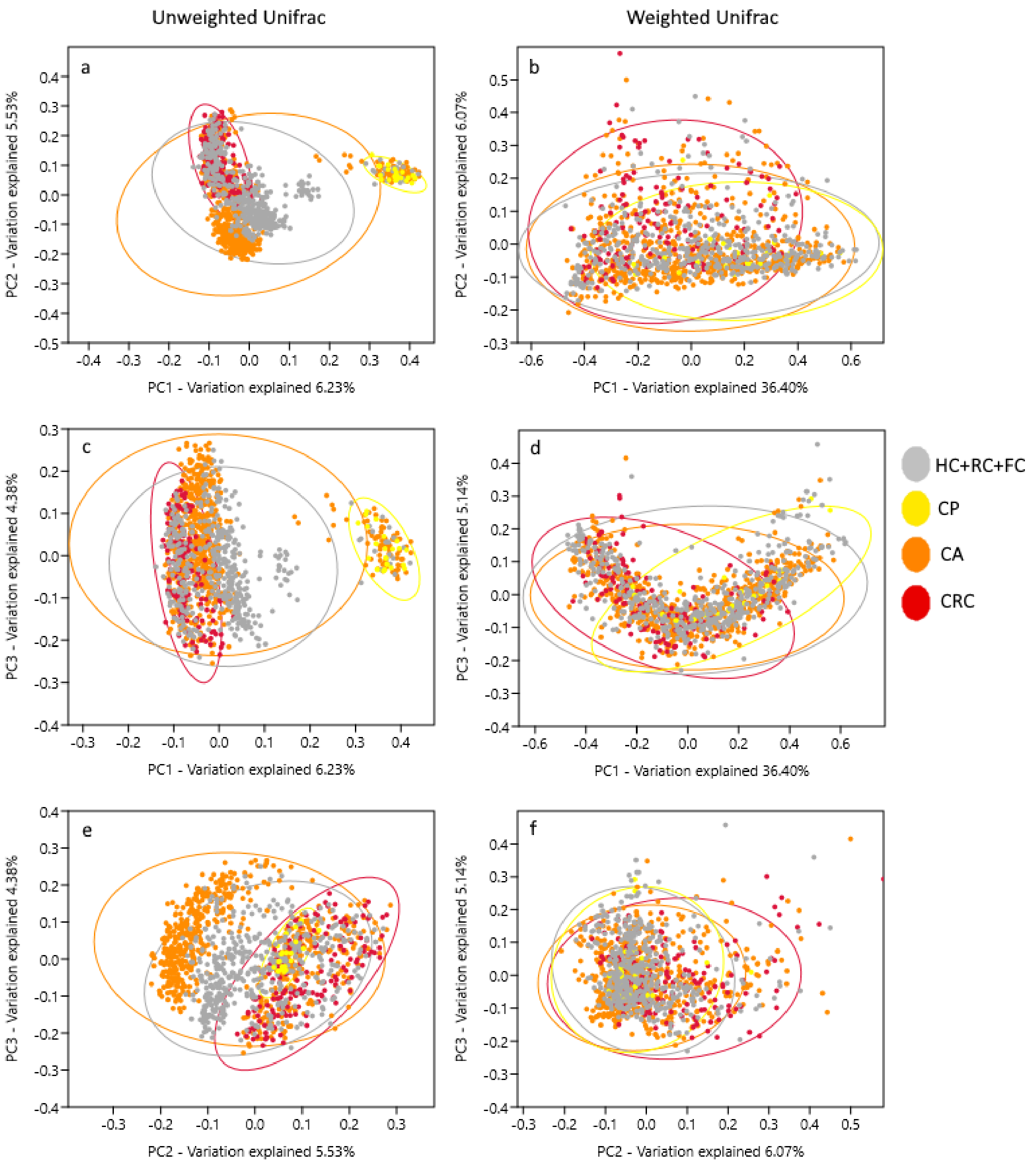

Figure S6. Beta diversity principal coordinates (PCoA) analysis of CRC-related groups. Comparison of healthy subjects (HC+RC+FC) and colon polyposis (CP), colon adenomatosis (CA) and colorectal cancer (CRC) patients. The Unifrac distance metric either unweighted (**a**, **c**, **e**) and weighted (**b**, **d**, **f**) was used. Ellipses indicate 95% confidence intervals.

Groups	Metric		DF	Squares sums	Mean squares	F.Model	R^2	Pr(>F)	Significance
**HC**+**RC**+**FC** vs. **MD** vs. **PD** vs. **WD**	Un-weighted unifrac	Groups	3	11.706	3.9019	16.484	0.125	0.001	
		Residuals	345	81.665	0.2367		0.875		
		Total	348	93.371			1		0.001 < p > 0
	Weighted unifrac	Groups	3	4.222	1.40732	10.685	0.085	0.001	
		Residuals	345	45.438	0.13171		0.915		
		Total	348	49.660			1		0.001 < p > 0
**HC** vs. **RC** vs. **FC**	Un-weighted unifrac	Groups	2	6.187	3.09359	13.072	0.056	0.001	
		Residuals	438	103.652	0.23665		0.944		
		Total	440	109.839			1		0.001 < p > 0
	Weighted unifrac	Groups	2	3.434	1.71706	11.988	0.052	0.001	
		Residuals	438	62.734	0.14323		0.948		
		Total	440	66.168			1		0.001 < p > 0
**HC**+**RC**+**FC** vs. **CP** vs. **CA** vs. **CRC** vs. **UC** vs. **CD**	Un-weighted unifrac	Groups	7	22.75	3.2498	13.58	0.066	0.001	
		Residuals	1356	324.5	0.2393		0.934		
		Total	1363	347.250			1		0.001 < p > 0
	Weighted unifrac	Groups	7	13.317	1.90242	13.586	0.066	0.001	
		Residuals	1356	189.882	0.14003		0.934		
		Total	1363	203.199			1		0.001 < p > 0

Table S11. Group significance for the Beta diversity PCoA determined by ADONIS method.

Groups	Metric	Sample size	Groups	Test statistic	p-value
**HC**+**RC**+**FC** vs. **MD** vs. **PD** vs. **WD**	Unweighted unifrac	349	4	0.49233	0.001
	Weighted unifrac	349	4	0.04007	0.024
**HC** vs. **RC** vs. **FC**	Unweighted unifrac	441	3	0.17641	0.001
	Weighted unifrac	441	3	0.02859	0.010
**HC**+**RC**+**FC** vs. **CP** vs. **CA** vs. **CRC** vs. **UC** vs. **CD**	Unweighted unifrac	1364	8	0.20897	0.001
	Weighted unifrac	1364	8	0.08381	0.001

Table S12. Group significance for the Beta diversity PCoA determined by ANOSIM method.

OTU	HC	RC	FC	MD	PD	WD	UC	CD	CP	CA	CRC
Firmicutes	58.9508	52.9856	50.5467	66.1773	62.6186	68.6163	63.7548	56.4517	43.9497	60.7098	57.1596
Bacteroidetes	27.2738	33.3556	44.5428	17.0162	27.3412	15.3187	27.6021	33.0409	50.2231	27.0028	23.5202
Actinobacteria	6.5485	5.69965	1.7042	11.0025	3.65092	11.9093	6.72538	3.4058	0.65232	1.27996	1.11119
Proteobacteria	3.47177	4.78957	1.14614	1.31932	4.4188	1.6484	1.29773	5.31665	3.84277	6.59364	9.79062
Verrucomicrobia	2.6703	2.57477	1.291	3.69522	1.49473	0.97642	0.32006	1.38157	0.00915	2.52138	3.95754
Euryarchaeota	0.58486	0.22516	0.29896	0.11895	0.05356	0.41173	0.03592	0.00063	0	1.08773	1.78846
Tenericutes	0.34588	0.10427	0.35122	0.04326	0.20936	0.77846	0.05546	0.01386	0.09713	0.36923	0.27315
Cyanobacteria	0.05804	0.1052	0.10017	0.3432	0.14885	0.25405	0.03963	0.01897	0.0536	0.06255	0.05791
Synergistetes	0.00977	0.05125	0.0014	0.06155	0.00696	0.00408	0.0001	0.0009	0.41287	0.08478	0.15758
Chloroflexi	0.00235	0.0342	0.00083	0.10815	0	0.01794	0.00085	0.00062	0.46939	0.02999	0.00108
Fusobacteria	0.01455	0.01969	0.00286	0.00241	0.00417	0.00848	0.13446	0.3524	0.01171	0.21451	2.04536
TM7	0.00828	0.01905	0.00554	0.08816	0.00696	0.00391	0.0304	0.01304	0.00128	0.00332	0.00137
Lentisphaerae	0.01327	0.00401	0.00547	0.00095	0.01182	0.02071	7.6E-05	0	0.00037	0.00599	0.01567
Acidobacteria	0.01871	0.00169	8E-05	3.4E-05	0	0.00098	3.8E-05	0.00012	0.00018	0.00215	0.00343
Spirochaetes	6.1E-05	3.1E-05	1.9E-05	0	0.03408	0	3.8E-05	5.8E-06	0	4.4E-05	0.05966
[Thermi]	0.02065	0.00217	1.3E-05	3.4E-05	0	0.00147	7.6E-05	1.2E-05	0.16756	0.01248	0
OP11	0	0.0197	0.0002	0.01366	0	0.00473	0	0	0.00073	0.00922	0.05252
Planctomycetes	0.0039	0.00093	0	0.00017	0	0	0	0	0.04866	0.0027	0
Gemmatimonadetes	0.00183	0.00246	0	0.00095	0	0.00098	0.00092	0.00156	0.0439	0.00216	0
Chlorobi	0.00047	9.3E-05	0.00155	3.4E-05	0	0.00799	0.00071	0.00026	0.00421	0.0013	0.00261
FBP	0.00016	0.00246	8E-05	0.00653	0	0.00228	0	0	0.00457	0.00011	0
Armatimonadetes	0	4.6E-05	0.00041	0.00071	0	0.00636	0	0	0.00018	0.00029	0.0003
ZB3	0	1.5E-05	0.0001	0	0	0.00228	0	1.2E-05	0	0.00042	0.00043
AD3	0	0	1.3E-05	0	0	0.00212	0	5.8E-06	0	1.9E-05	0
Elusimicrobia	0.00028	0.00129	0	0	0	0	0.0005	0.00019	0.00037	0.00184	4.8E-05
GN02	0	0	4.5E-05	0	0	0.00179	3.8E-05	3.5E-05	0	4.4E-05	0
Nitrospirae	0.00075	0.00026	0	6.8E-05	0	0	0.00032	0.00027	0.00146	0.00014	0
TM6	0.00022	0.00011	0	0	0	0	8.9E-05	0	0	0	0.00107
Deferribacteres	0.00022	4.6E-05	0	0	0	0	0	5.8E-06	0.00091	0.00013	1.1E-05
Fibrobacteres	0.00022	0	3.5E-05	0	0	0	0	0	0.00073	0	0
Crenarchaeota	0.00017	0	0	0	0	0	3.8E-05	8.7E-05	0.00055	1.6E-05	0
SR1	0	0.00022	0	0	0	0	0	0	0.00055	0.0004	0
WPS-2	2.4E-05	0.00017	0	3.4E-05	0	0	0.00011	0.00011	0.00055	0.0001	0
p__	1.2E-05	0	2.6E-05	0.00048	0	0.00016	0	5.8E-06	0	5.9E-05	0
BRC1	8.5E-05	0	9.6E-06	0	0	0	5.1E-05	5.2E-05	0.00037	2.8E-05	0
OD1	2.4E-05	6.2E-05	2.2E-05	0	0	0.00016	2.5E-05	7E-05	0.00037	9.4E-06	0
SAR406	0	9.3E-05	0	0	0	0	1.3E-05	5.2E-05	0.00037	5.9E-05	0
WS3	2.4E-05	4.6E-05	0	0	0	0	1.3E-05	5.2E-05	0.00018	0.00033	0.00018
Thermotogae	0	0	5.7E-05	0	0	0	5.1E-05	2.9E-05	0.00018	1.3E-05	0
Caldiserica	0	0	1.9E-05	0	0	0.00016	0	0	0	6.3E-06	0
MVS-104	0	0	0	0.0001	0	0	0	0	0	0	0
AC1	0	0	2.6E-05	0	0	0	0	0	0	5.9E-05	3.2E-05
NC10	0	0	2.2E-05	0	0	0	0	0	0	5.6E-05	0
H-178	0	0	0	3.4E-05	0	0	0	0	0	0	0
GOUTA4	0	1.5E-05	0	0	0	0	0	0	0	3.1E-06	3.2E-05
OP3	0	1.5E-05	0	0	0	0	0	0	0	2.8E-05	0
WWE1	0	1.5E-05	0	0	0	0	0	0	0	2.2E-05	0
WS1	0	0	9.6E-06	0	0	0	0	0	0	1.6E-05	0
TPD-58	0	1.5E-05	0	0	0	0	0	5.8E-06	0	3.1E-06	1.1E-05
OP1	1.2E-05	0	0	0	0	0	0	5.8E-06	0	1.3E-05	0
LCP-89	0	0	0	0	0	0	0	0	0	9.4E-06	0
NKB19	0	0	0	0	0	0	0	0	0	9.4E-06	0
OP8	0	0	6.4E-06	0	0	0	0	0	0	3.1E-06	0
WS5	0	0	6.4E-06	0	0	0	0	0	0	6.3E-06	0
GN04	0	0	0	0	0	0	0	0	0	6.3E-06	0
SC4	0	0	0	0	0	0	0	0	0	6.3E-06	0
VHS-B3-43	0	0	0	0	0	0	0	0	0	6.3E-06	0
Chlamydiae	0	0	3.2E-06	0	0	0	0	0	0	3.1E-06	0
OP9	0	0	0	0	0	0	0	0	0	3.1E-06	0

Table S13. Average OTU composition (%) at phylum level.
